# Supplementary material for: Gallic Acid: A Natural Phenolic Compound Exerting Antitumoral Activities in Colorectal Cancer via Interaction with G-Quadruplexes
Source: Cancers (Basel). 2022 May 26;14(11):2648. doi: 10.3390/cancers14112648 (PMC9179882; doi:10.3390/cancers14112648)
Supplement: Supplementary file 1 [file cancers-14-02648-s001.zip › cancers-1732904-supplementary.pdf]

cancers

MDPI

Supplementary Materials

Gallic Acid: A Natural Phenolic Compound Exerting Antitumoral Activities in Colorectal Cancer via Interaction with G-Quadruplexes

Victoria Sanchez-Martin[1,2,3], María del Carmen Plaza-Calonge[1], Ana Soriano-Lerma[1,4], Matilde Ortiz-Gonzalez[1,5], Angel Linde-Rodriguez[1,2], Virginia Perez-Carrasco[1,2], Inmaculada Ramirez-Macias[1,2], Marta Cuadros[1,3], Jose Gutierrez-Fernandez[2], Javier Murciano-Calles[6], Juan Carlos Rodríguez-Manzaneque[1], Miguel Soriano[1,5,†] and Jose Antonio Garcia-Salcedo[1,2,*,†]

Table S1. List of all antibodies and respective dilutions used for immunofluorescence (IF) and western blot (WB) experiments in this study.

Name	Comercial Reference	Study	Dilution
BG4	NA	IF	20 nM
		WB	-
Mouse α-nuclelolin	Invitrogen (39-6400)	IF	1:100
		WB	-
Rabbit α-fibrillarin	Abcam (ab5821)	IF	1:100
		WB	-
Mouse α-POLR1A	Santa Cruz Biotechnology (sc-48385)	IF	1:100
		WB	1:100
Mouse α-FLAG	Sigma Aldrich (F1804)	IF	1:1000
		WB	-
Rabbit α-gH2AX	Cell Signaling Technology (9718)	IF	-
		WB	1:800
Mouse α-actin	Sigma Aldrich (A5441)	IF	-
		WB	1:5000
Goat α-mouse Alexa Fluor 488	Invitrogen (A-11001)	IF	1:500
		WB	-
Donkey α-rabbit Alexa Fluor 555	Invitrogen (A-31572)	IF	1:1000
		WB	-
Goat α-mouse HRP conjugated	Promega (W4021)	IF	-
		WB	1:2500
Donkey α-rabbit HRP conjugated	Invitrogen (SA1-200)	IF	-
		WB	1:2500

Table S2. List of all forward (FW) and reverse (RV) primers and respective sequences used for qRT-PCR in this study. Supplementary.

Name		Sequence (5′->3′)
5′ETS	FW	GTGCGTGTCAGGCGTTCT
	RV	GGGAGAGGAGCAGACGAG
ACTIN	FW	TGCGTCTGGACCTGGCTGGC
	RV	GCCTCAGGGCAGCGGAACCG
BCL2	FW	CTGCACCTGACGCCCTTCACC
	RV	CACATGACCCCACCGAACTCAAAGA
CMYB	FW	ACCATGACTATGATGGGCTGC
	RV	TCCCCAAGTGACGCTTTCC
CMYC	FW	CGTCCTCGGATTCTCTGCTC
	RV	GCCTGCCTCTTTTCCACAGA
KRAS	FW	GACTGAATATAAACTTGTGGTAGTTGGA
	RV	CATATTCGTCCACAAAATGATTCTG
VEGFA	FW	CTACCTCCACCATGCCAAGT
	RV	GCAGTAGCTGCGCTGATAGA

Table S3. Sequences of G4-containing oligonucleotides used for fluorescence intercalator displacement assay, PCR-stop assay, circular dichroism experiments and UV-visible spectroscopy.

		**G4-oligonucleotides for FID Assay**
Name		Sequence (5′->3′)
5′ETS		GGGGGGCGGGTGGTTGGG
BCL2		TAGGGGCGGGCGCGGGAGGAAGGGGGCGGGAGCGGGGCTG
CMYB		TAGGAGGAGGAGGTCACGGAGGAGGAGGAGAAGGAGGAGGAGGAAA
CMYC		GGGGCGCTTATGGGGAGGGTGGGGAGGGTGGGGAAGGTGGGGAGGAG
KRAS		TAGGGCGGTGTGGGAAGAGGGAAGAGGGGGAGGCAG
VEGFA		GGGGCGGGCCGGGGGCGGGG
TEL		TAGGGTTAGGGTTAGGGTTAGGGT
		**G4-oligonucleotides for PCR-stop assay**
Name		Sequence (5′->3′)
5′ETS	FW	TCGCGTGGGGGGCGGGTGGTTGGG
	RV	TTCTCGTCCCAACCAC
CMYC	FW	GGGGCGCTTATGGGGAGGGTGGGGAGGGTGGGGAAGGTGGGGAGGAG
	RV	TTCTCGTCTCCTCCCC
		**G4-oligonucleotides for CD spectra**
Name		Sequence (5′->3′)
5′ETS		GGGGGGCGGGTGGTTGGG
CMYC		TGGGGAGGGTGGGGAGGGTGGGGAAGG
		**G4-oligonucleotides for UV-vis spectra**
Name		Sequence (5′->3′)
5′ETS		GGGGGGCGGGTGGTTGGG
CMYC		TGGGGAGGGTGGGGAGGGTGGGGAAGG

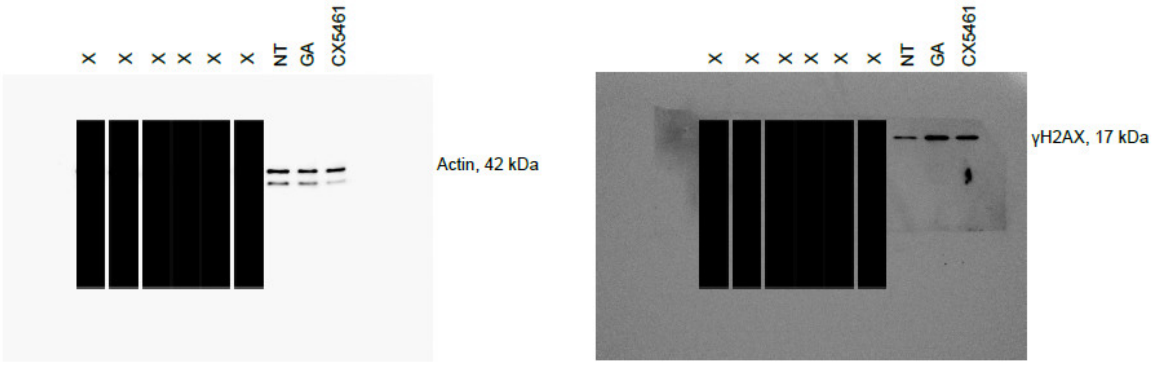

Densitometry readings

	NT	GA	CX5461
Actin	42,230.773	32,234.409	38,869.723
γH2AX	11,712.208	38,832.957	44,721.522
γH2AX/Actin	0.2773	1.2047	1.1505

Figure S1. Uncropped Western blot in SW480 cells upon treatment with vehicle (NT), GA IC_{50} or CX5461 10 μM for 6 h to determine protein levels of γH2AX as a marker of DNA damage and actin as housekeeping gene. Supplementary

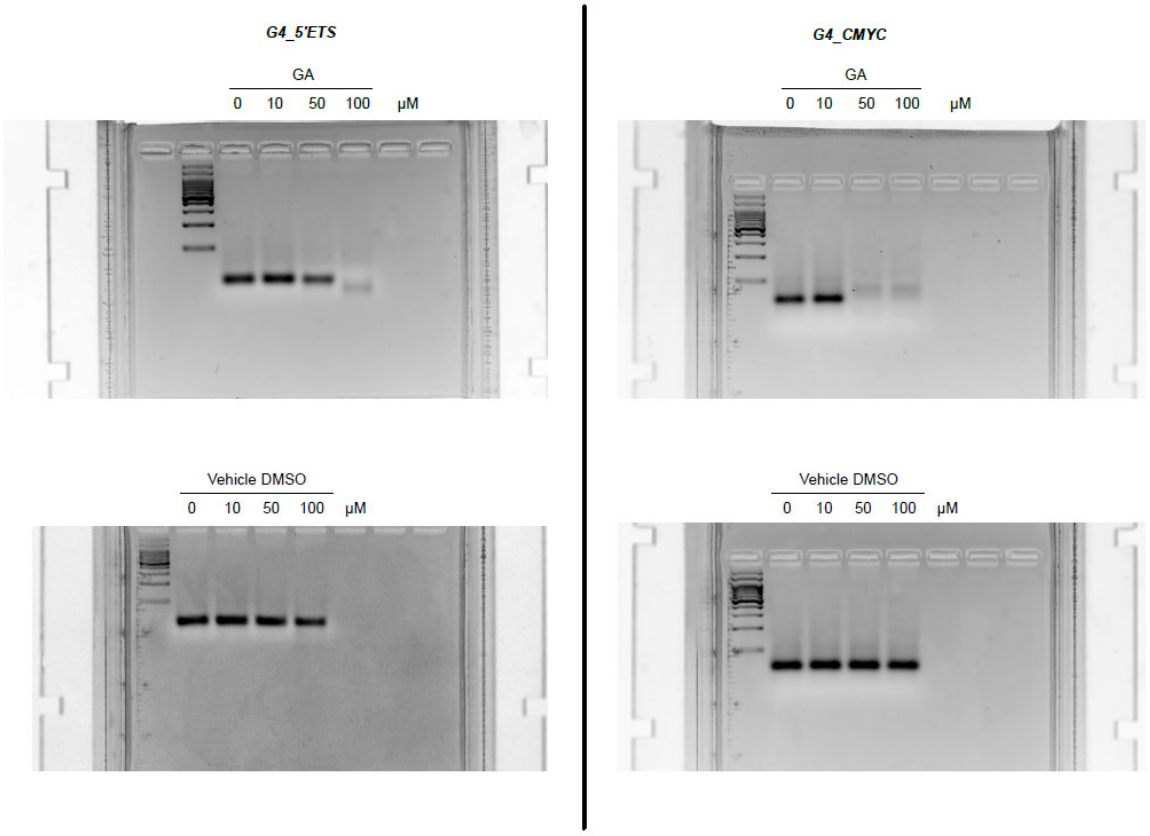

Figure S2. Uncropped gel from a PCR-stop assay including the G4-containing oligonucleotide of *5′ETS* and *CMYC* with increasing concentrations of GA or the corresponding vehicle DMSO.

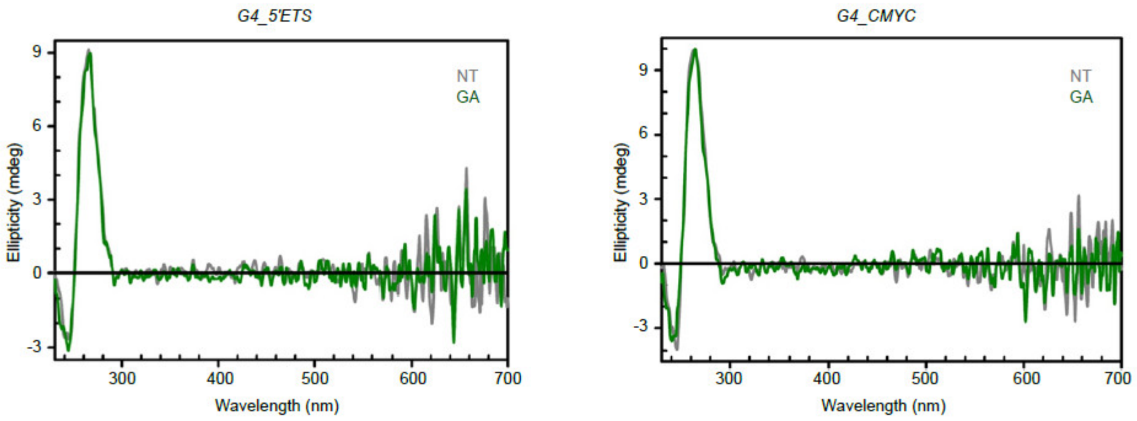

Figure S3. Full CD spectra of the G4s formed by *5′ETS* and *CMYC* in the absence (gray) or presence (green) of GA 100 μM.
